# Mifepristone combined with ethacridine lactate for third-trimester stillbirth induction: a 5-year experience from Shanghai

**DOI:** 10.1186/s12884-022-05104-0

**Published:** 2022-10-26

**Authors:** Rui-Hong Xue, Juan Li, Yong-Li Yao, Run-Jie Huang, Jue Ma, Lin Zhang

**Affiliations:** 1 The International Peace Maternity and Child Health Hospital, School of Medicine,Shanghai Jiao Tong University, Shanghai, China; 2 Shanghai Key labouratory of Embryo Original Diseases, 200030 Shanghai, China; 3 Institute of Birth Defects and Rare Diseases, School of Medicine, Shanghai Jiao Tong University, 200030 Shanghai, China

**Keywords:** Ethacridine lactate, Mifepristone, Stillbirth, Induction, Third-trimester

## Abstract

**Objective:**

To review and analyze the efficacy and safety of mifepristone combined with ethacridine lactate for induction of stillbirth in the third trimester.

**Methods:**

All patients with stillbirth in late pregnancy (≥ 28 weeks) in a university-affiliated maternity center from October 2016 to September 2021 were included in this study. After exclusion, patients were divided into ethacridine lactate and non-ethacridine lactate groups according to induction methods. Logistic regression was conducted to identify the risks of complications.

**Results:**

We identified 122 patients that experienced stillbirth (5’ Apgar score = 0) in third-trimester from the 5-year total deliveries in the hospital, among whom 39 stillbirths that resulted from termination of pregnancy for severe fetal anomalies and 1 stillbirth that was in twin pregnancy were excluded. Thus, 82 cases with stillbirths (dead before induction) were included in the analyses. In the 82 cases, 49 (59.76%) accepted intra-amniotic ethacridine lactate induction with 47 (95.92%, 47/49) successfully induced. No statistical difference was observed in induction failure rate between ethacridine dosage groups of < 75mg and ≥ 75mg (0/25, vs. 2/24, respectively; P > 0.05). The ethacridine lactate induction group showed no increased risks in complications (6.12%, 3/49), compared with non-ethacridine lactate group (12.12%, 4/33) (P = 0.35, OR, 0.47, 95%CI, 0.10 to 2.27).

**Conclusion:**

Mifepristone combined with ethacridine lactate is a safe and low-risk induction method for patients with stillbirth in the third trimester.

## Background

Stillbirth is defined as the death of a fetus in utero at a gestational age ≥ 20 weeks or at a weight ≥ 500g. Stillbirth is a rare yet devastating complication for pregnant women. Globally in 2019, an estimated 2?0 million babies were still born at 28 weeks or more of gestation, with a global stillbirth rate of 13?9 stillbirths per 1000 total births [1]. The stillbirth rate was 13.2 per 1000 births in China in 2015–2016, and nearly one-third of all stillbirths may be preventable [2]. In 2016, based on data between 2012 and 2014 from the China National Maternal Near Miss Surveillance System, Zhu et al. calculated a third-trimester stillbirth rate of 8.8 per 1000 births [3]. The global burden of stillbirth has far-reaching psychosocial impacts on women, families, caregivers and communities and a wide-ranging economic impact on health systems and society [4]. The Lancet 2011 Stillbirth Series Call to Action brought attention to stillbirth as a neglected global public health issue [5, 6]. However, the Lancet 2016 series revealed that little real progress has been made since the 2011 Call to Action. For parents, stillbirth is a devastating complication. They usually experience pain and depression. How to provide relief for these parents with a convenient and safe induction method has important clinical significance.

For pregnancy termination in the second trimester, mifepristone combined with ethacridine lactate may shorten the induction-to-abortion time compared with the use of ethacridine lactate alone, without increasing the number of complications [7]. The induction-to-abortion interval, blood loss over 24h, the rate of retained placental tissue and uterine evacuation were significantly lower in the mifepristone combined with ethacridine lactate group than in the ethacridine lactate alone group in second-trimester pregnancy termination, without increasing complications and side effects apart from nausea [8]. The simplicity of the technique and equipment required to perform ethacridine lactate instillation in second-trimester pregnancy termination is a significant advantage. Another advantage of ethacridine lactate is that it can be used safely in patients with cardiovascular and renal diseases [9], and ethacridine lactate appears to be a safe and effective agent for pregnancy termination during the 2nd trimester. Mifepristone combined with ethacridine lactate is safe and effective for patients with low placentation or/and prior cesarean section for pregnancy termination in the second trimester [10, 11].

For pregnancy termination in the third trimester, intra-amniotic injection of ethacridine lactate demonstrated good clinical effects and could be used as a suitable method for pregnancy termination in the third trimester in women with prior cesarean Sect [12]. However, in the absence of evidence for stillbirth induction, recommendations are made based on clinicians’ knowledge and experiences and further research is needed [13].

For stillbirth in the third trimester, the literature on ethacridine lactate is very limited, and the safety and effectiveness of ethacridine lactate in the induction of stillbirth in the third trimester remains unclear. In this article, we sought to investigate the safety and efficacy of intra-amniotic ethacridine lactate for stillbirth induction in the third trimester, which may provide us with a convenient and simple induction method in the clinic for stillbirth in the third trimester.

## Materials and methods

A 5-year retrospective study was conducted to ascertain the induction methods regarding stillbirth in third-trimester pregnancy. After obtaining written informed consent and approval from the local ethics committee, we used data from the digital medical records system of the hospital. Women who had been diagnosed with **s**tillbirth (5’ Apgar score = 0) in the third trimester (≥ 28 gestational weeks) between October 2016 and September 2021 were included. Stillbirths that resulted from the termination of pregnancy for severe fetal anomalies (5’ Apgar score = 0, but the fetus was alive before induction) and twin pregnancy stillbirths were excluded. After exclusion, patients were divided into ethacridine lactate and non-ethacridine lactate groups according to induction methods.

After admission, information on patients who had suffered from stillbirth in the third trimester was collected, including demographic variables, medical and obstetric history, and labouratory values, and these patients underwent labouratory and physical inspections, including blood tests, leucorrhoea examinations, and electrocardiograms. Women who did not have threatened labour or ruptured membranes underwent cervical examination. After excluding patients with contraindications, such as those with abnormal liver function and drug allergies, the cervical maturity degree (Bishop score) was the main consideration in suggesting the induction method. When the cervix is favorable, some doctors might prefer to choose artificial amniotomy (+ oxytocin). When the cervix is unfavorable, different doctors might suggest different induction methods. Some doctors might suggest misoprostol according to the guideline recommendations, while some doctors might suggest the intra-amniotic injection of 50–100mg of ethacridine lactate for stillbirth induction because ethacridine lactate had been traditionally used in the second trimester pregnancy termination in our hospital for many years, and the ethacridine lactate used in stillbirth induction in the third trimester had been proven to be safe according to their clinical experiences. Thus, it is urgent to summarize these experiences as a conclusion to guide clinical practice.

In our hospital, ethacridine lactate (yellow colour), with the lowest dose of 50mg (2 ml per bottle, usually using 3 ml, 1.5 bottles), was diluted with sterile water (3 ml each bottle) for injection. The doctors performed intra-amniotic ethacridine lactate injection guided by B-ultrasound with a syringe (10 ml) and long needle (9 G/900 mm). When the needle arrived in the intra-amniotic before or under ultrasonic localization, the doctor pulled back the syringe, and amniotic fluid was pumped back, which indicated the right depth. Then, intra-amniotic diluted ethacridine lactate injection was performed. After the injection of ethacridine lactate, most women with stillbirth delivered approximately 36h later. During this waiting time, mifepristone (100mg, qd., Po*2d) was administered to promote cervical maturation in almost all women.

This study was performed in accordance with the Declaration of Helsinki, and ethical approval for this study was granted by the ethics committee on human research at the International Peace Maternity and Child Health Hospital, Shanghai, China on August 15, 2020 (Review Board: (GKLW) 2020 − 113).

### Study population

The International Peace Maternity and Child Health Hospital is one of the largest maternity hospitals in China; every year, there are 10 000 to 15 000 low- and high-risk deliveries. It is one of five Shanghai prenatal diagnosis centres. Patients come from Shanghai and some provinces of China. The stillbirth rates have ranged from 1–3‰ in different regions of Shanghai in recent years. Women who were diagnosed with **s**tillbirth (5’ Apgar score = 0) in the third trimester (≥ 28 gestational weeks) between October 2016 and September 2021 were included. Stillbirths that resulted from the termination of pregnancy for severe anomalies (5’ Apgar score = 0, but the fetus was alive before induction) and twin pregnancy stillbirths were excluded. After exclusion, patients were divided into ethacridine lactate and non-ethacridine lactate groups according to induction methods.

### Outcomes

In the clinic, after the 28th gestational week, women who were conscious of decreased fetal movement and came to the hospital underwent B-ultrasound scanning, and if there was no fetal heartbeat, the diagnosis of stillbirth was made. The data of patients in our study were exported through the digital medical records system of the hospital. We retrospectively defined a 5’ Apgar score = 0 as the origination of preliminary stillbirth data, and stillbirths that resulted from the termination of pregnancy for severe fetal anomalies (5’ Apgar score = 0, but the fetus was alive before induction) and twin pregnancy stillbirths were excluded from our final analyses. Induction failure was defined as an induction process exceeding 72h. The complications mainly indicated patients with hemorrhage, injury, hematuria, or other recorded adverse outcomes for maternity, such as the induction-to-delivery interval, blood loss over 24h, the rate of retained placental tissue and uterine evacuation. Postpartum hemorrhage was defined as more than 500 ml with the weighing method. Injuries indicated adverse injury outcomes related to induction or labour. hematuria indicated gross hematuria during labour.

### Analysis

During our study period, different induction methods were reviewed, and the proportional rates were calculated and compared. Non-parametric distributions were described with median and interquartile range (25th percentile − 75th percentile) in numeric variables. Data analysis was conducted using logistic regression to identify the risks of induction failure between different ethacridine lactate dosage groups. Furthermore, the risks of complications were assessed using logistic regression to identify whether the ethacridine lactate induction group had increased risks of adverse complications compared with the non-ethacridine lactate group. A P value < 0.05 was considered statistically significant. Data analysis was conducted using the Statistical Package for Social Sciences version 23.0 (SPSS, IBM, USA).

## Results

**Fig. 1:**
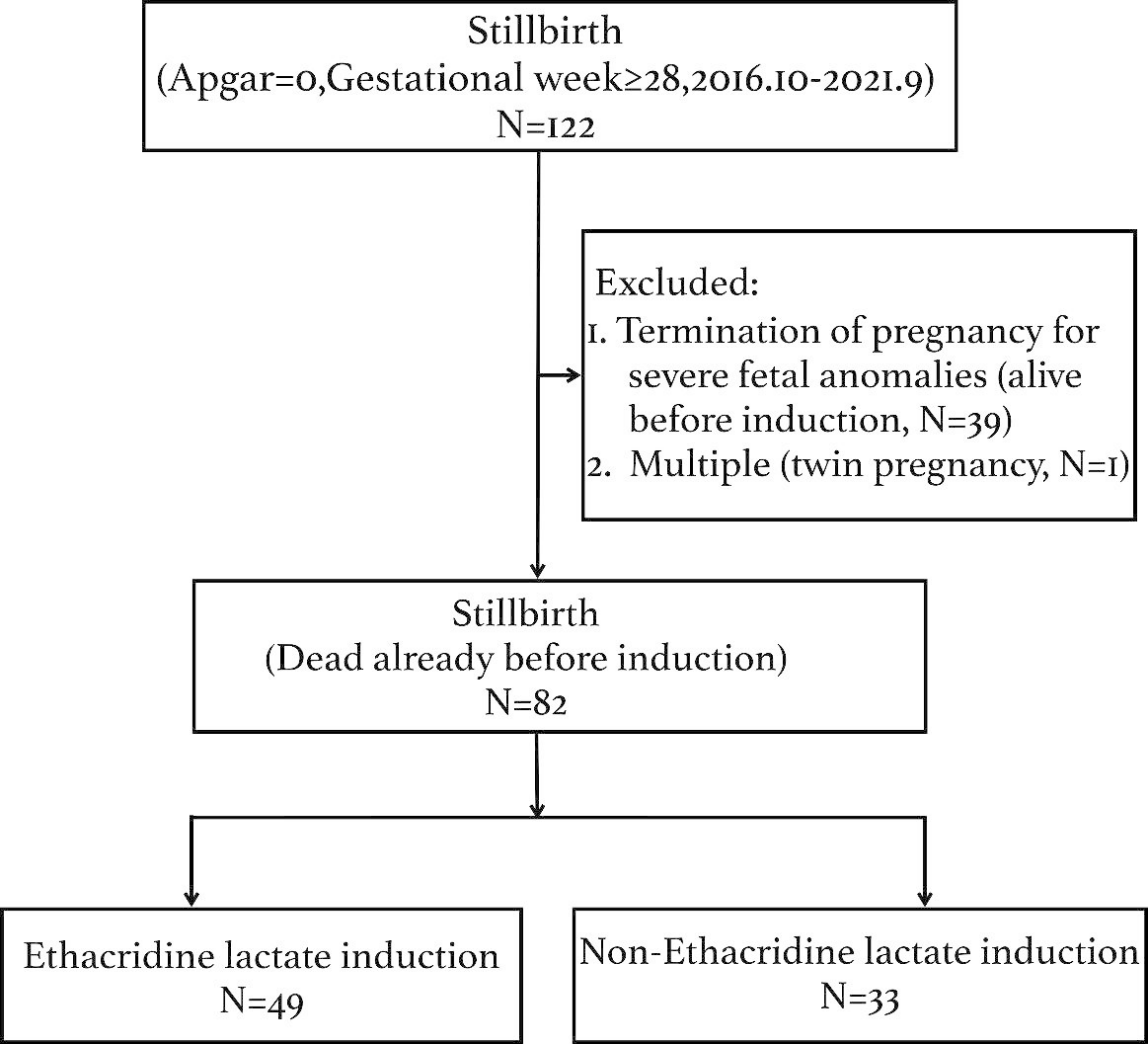
The flowchart of patient inclusion of the study

By retrospectively studying the patients’ digital records between October 2016 and September 2021, 122 women were diagnosed with **s**tillbirth (5’ Apgar score = 0) in the third trimester (≥ 28 gestational weeks). Thirty-nine stillbirths that resulted from the termination of pregnancy for severe fetal anomalies (5’ Apgar score = 0, but the fetus was alive before induction) and one twin pregnancy stillbirth were excluded. Ultimately, 82 women with stillbirths were included in our final analyses, including 49 women in the ethacridine lactate group and 33 in the non-ethacridine lactate group (Fig. 1).

Among the 82 women with stillbirths, the median (25th -75th percentile) of maternal age was 31 (28.75–35) years, the gestational week was 33.2 (30.05–35.95) weeks, and the induction length was 30 (22–37) hours, the parity was 1 (1-1.25). Among the 82 women with stillbirths, eleven women (13.41%, 11/82) had prior cesarean section, among whom 9 received ethacridine lactate (one patient was diagnosed with induction failure and had 630 ml of blood loss), and 2 women had spontaneous labour. Specifically, 49 (59.76%, 49/82) women received ethacridine lactate (combined with oral mifepristone; only 3 women received ethacridine lactate injection without oral mifepristone, but they all had successful deliveries, with 36/37/31 hours from the injections to deliveries) induction, among whom 2 patients were diagnosed with induction failure, and the effective rate of ethacridine lactate for patients with stillbirth in the third trimester was 95.92% (47/49).

Table 1 shows the clinical characteristics of the women with stillbirths in the study. Among the 82 women with stillbirths, the median (25th -75th percentile) of maternal age was 31 (28.75–35) years, the gestational week was 33.2 (30.05–35.95) weeks, and the induction length was 30 (22–37) hours, the blood loss was 250 (170–307.5) ml, and the stillbirth weight was 2145 (1215–2565), and the parity was 1 (1-1.25) (Table 1).

**Table 1:** Clinical characteristics of stillbirth in the study period

Characteristics	Median (25th -75th percentile)	
Maternal age(years)	31 (28.75–35)	
Gestational week	33.2 (30.05–35.95)	
Induction interval(h)	30 (22–37)	
Hemorrhage(ml)	250 (170–307.50)	
Stillbirth weight(g)	2145 (1215–2565)	
Parity	1 (1-1.25)	

**Fig. 2:**
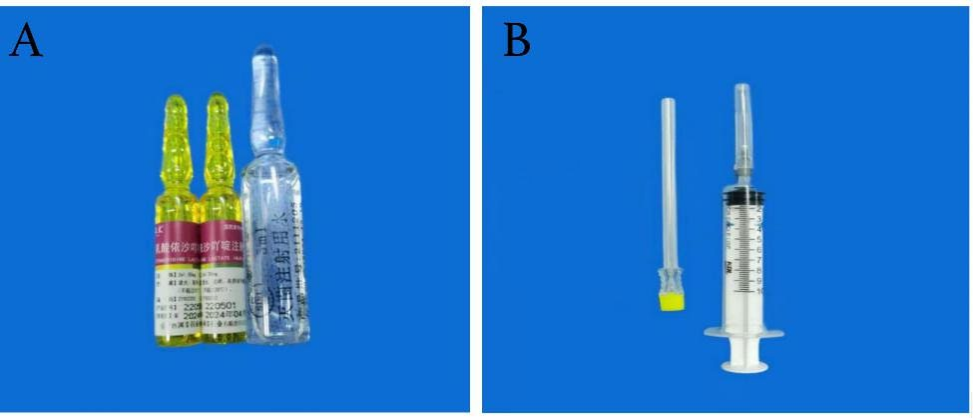
The pictures of ethacridine lactate, the syringe and the needle

Figure 2 shows the ethacridine lactate (yellow colour), with the lowest dose 50mg (2 ml per bottle, usually using 3 ml, 1.5 bottles), which could be diluted with sterile water (3 ml each bottle) for injection (Fig. 2A). The doctors performed intra-amniotic ethacridine lactate injection guided by B-ultrasound with a syringe (10 ml) and long needle (9 G/900 mm) (Fig. 2B).

Table 2 shows the methods of third-trimester stillbirth induction; 59.76% (49/82) of the women underwent ethacridine induction (Table 2). Table 3 shows the induction methods at different gestational weeks and their efficacy. In this table, stillbirths with different induction methods were divided according to gestational weeks. In the ethacridine method, 20 patients (one with 560 ml of blood loss) were included in the 28–31 weeks groups, 23 patients (one with failure and 630 ml of blood loss) were included in the 32–36 weeks group, and 6 patients (one who suffered from hematuria during labour) were included in the ≥ 37 weeks group. The overall effective rate was 95.92% (47/49). The duration length(hours) from induction to delivery was presented for every induction method and gestational group (Table 3).

**Table 2:** Methods of third-trimester stillbirth induction

Total(n)	Methods	n(%)
N = 82	Ethacridine (& Mifepristone)	49(59.76)
	Oxytocin	8(9.76)
	Misoprostol + Mifepristone	6(7.32)
	Intravaginal misoprostol only	5(6.10)
	Artificial amniotomy + Oxytocin	4(4.88)
	Mifepristone only	1(1.22)
	Oxytocin + balloon + Mifepristone	1(1.22)
	Balloon + Artificial amniotomy + Mifepristone	1(1.22)
	Spontaneous labour	7(8.54)

**Table 3:** Induction methods in different gestational weeks and their efficacy

Methods	28-31week			32-36w			>=37			
	Cases (n)	Duration^a^, Median (25th -75th percentile)	Complication(n)	Cases (n)	Duration^a^, Median (25th -75th percentile)	Complication (n)	Cases (n)	Duration^a^, Median (25th -75th percentile)	Complication (n)	
Ethacridine(&Mifepristone)	20	31 (25–36)	1 (hemorrhage 560ml)	23	32(29-42.50)	1 (hemorrhage 630ml)	6	37 (31.50–50)	1 (hematuria)	
Oxytocin	1	4(4–4)	0	2	35(31–39)	0	5	25 (25–30)	1 (hemorrhage 620ml)	
Misoprostol + Mifepristone	4	16.50(12.25–25.25)	0	2	28(26.50–29.50)	0	0	-	0	
Intravaginal misoprostol only	3	13(11–19)	0	2	36.50(31.75–41.25)	0	0	-	0	
Artificial amniotomy(+ Oxytocin)	1	4.50(4.50–4.50)	1 (hemorrhage 850ml for placental abruption)	0	-	0	3	9.50(5-24.25)	2 (one rectal injury for macrosomia; one hemorrhage 1500ml for placental abruption)	
Mifepristone only	0	-	0	1	15(15–15)	0	0	-	0	
Oxytocin + Balloon + Mifepristone	0	-	0	1	14(14–14)	0	0	-	0	
Balloon + Artificial amniotomy + Mifepristone	0	-	0	0	-	0	1	32(32–32)	0	
Spontaneous labour	0	-	0	5	-	0	2	-	0	

^a^ **Duration (hours): the median(25th-75th percentile) duration hours from induction to delivery**

Table 4 shows the comparation of different ethacridine dosages in third-trimester stillbirth induction. Patients were divided into two groups, the < 75mg group and the ≥ 75mg group, according to ethacridine dosage. Among the 49 women treated with ethacridine, 25 women were included in the < 75mg group, and 24 women were included in the ≥ 75mg group. The induction failure rates were compared with statistical methods, and no statistical significance was found between the different dosage groups (P > 0.05) (Table 4).

**Table 4:** Comparation of different ethacridine dosages in third-trimester stillbirth induction

Total	Ethacridine Dose	n(%)	Interval (h)	Week	Hemorrhage (ml)	Failed, n,(%)	Groups	Failed, n	P
N = 49	< 75mg	25 (51.02)	35.56	34.06	271.4	0	A(28-31w): n = 6	0	> 0.05
							B(32-36w):n = 14	0	
							C( > = 37):n = 5	0	
	≥ 75mg	24 (48.98)	35.38	31.11	211.88	2 (8.33)	A(28-31w): n = 14	1	
							B(32-36w):n = 9	1	
							C( > = 37):n = 1	0	

Table 5 shows the complications of the different induction methods. In the ethacridine lactate group, 2 women had hemorrhage (≥ 500 ml), and one had gross hematuria; the complication rate was 6.12% (3/49). In the non-ethacridine lactate group, 3 women had hemorrhage (≥ 500 ml), and one had rectal injury; the complication rate was 12.12% (4/33). After logistic regression, no statistical significance was found in adverse complications between the ethacridine lactate group and the non-ethacridine lactate group (P = 0.35, OR, 0.47, 95% CI, 0.10 to 2.27) (Table 5).

**Table 5:** Complications in different induction methods

		Hemorrhage > 500ml,n, (%)	Hematuria, n	Rectal injury, n	Total, n, (%)	P value	Odds ratio (95% CI)
N = 82	Ethacridine N = 49 (59.76%)	2 (3.92)	1	0	3 (6.12)	0.35	0.47(0.10,2.27)
	Non-Ethacridine N = 33 (40.24%)	3 (9.09)	0	1	4 (12.12)		

In our study, when the induction process exceeded 72h, which was considered induction failure, further measures were taken. There were 2 patients diagnosed with induction failure, and they were all in the mifepristone combined with ethacridine lactate group, which included women at 32 weeks plus 1day and 28 weeks plus 2 days of gestation. In these two patients, one patient with failure subsequently received an oxytocin intravenous drip and delivered the next day, and the other patient received intravaginal misoprostol (400mg) twice and delivered.

## Discussion

In this retrospective study, we found that mifepristone combined with ethacridine lactate accounts for the majority of stillbirth inductions in the third trimester in our hospital and is a safe and low-risk induction method for patients with third-trimester stillbirths.

Ethacridine lactate, as an old medication used for second trimester pregnancy induction, has been widely used in China for many years due to its low cost, convenience, safety, high successful induction rate (> 95%) and lack of pain for patients and is also suitable for pregnancy termination in patients with prior cesarean section. However, ethacridine lactate for third-trimester stillbirth induction has rarely been reported in the foreign literature, let alone in guideline recommendations.

Overcoming unfavorable cervix, however, remains the most challenging task in the induction of labour. It has been reported [14] that combined mifepristone-misoprostol could resulted in shorter induction-to-delivery intervals for the second trimester induce of labour(IOL), compared with the using of sulprostone, and in this study, they also reported cases of third-trimester stillbirth induction, whey all received misoprostol alone. Until now, the current literature on mifepristone-misoprostol using in the third trimester remains limited, compared with that in the second trimester, and few studies specified the actual number of patients in the third trimester, and future research is necessary on third-trimester outcomes with mifepristone-misoprostol [15].

In our hospital, ethacridine lactate for third trimester stillbirth induction has been used for several years in clinical practice. Compared with the international guidelines, one reason why misoprostol is not widely used in China might be the lack of low-dose misoprostol because it is considered that high-dose misoprostol might cause uterine rupture. Previously, the lowest dosage of misoprostol was 0.2mg (200µg, per tablet) in our hospital. Until now, the lowest dosage of misoprostol, 25µg, has been available in our hospital. In fact, during our study period, the methods of third-trimester stillbirth induction had no unified consensus by different doctors from different obstetric wards in our hospital, and they might suggest different induction methods for stillbirth in the third trimester, even under similar cervical circumstances.

The use of intracervical Foley’s catheter is popular in resource-constrained settings [16]. However, in our hospital, Foley’s catheter or a Cock balloon was used preferentially in women after induction failure for the first time. Artificial amniotomy alone or combined with oxytocin might be used when the cervix is favorable, as reported [17]. Consequently, the development of safe and cost-effective agents to ripen the cervix and induce contractions remains a major research issue [18]. Previously, formulations of prostaglandin E2 (PGE2) administered vaginally were accepted as the gold standard for labour induction [19]. As the guideline recommended, in the event of intrauterine fetal death, if there is evidence of ruptured membranes, infection or bleeding, immediate induction of labour or cesarean birth should be performed. If a woman chooses an induced labour, oral mifepristone (200mg) followed by vaginal dinoprostone or oral or vaginal misoprostol, or a mechanical method of induction could be performed [13].

The reason why we excluded the termination of pregnancy for severe fetal anomalies was not because these patients had more complications. Fetal kill by injection of potassium chloride before induction was not wildly accepted in China for abortion. Baby were normally alive in the uterus before induction for the termination of pregnancy for fetal anomalies, while in our paper, we focused only on the safety and efficacy of third-trimester stillbirth (the baby was dead before induction). The exclusion of patients with termination of pregnancy for severe fetal anomalies was to reduce the statistical bias; for example, the comparison of induction-to-delivery intervals between different groups might be difficult when confronted with fetal demise or not. It is unclear whether the changes of maternal-fetal hemodynamic and placenta function have somewhat effects on the drug effects and consequently affect the induction intervals after the occurrence of a fetus death in utero, and it needs further investigations.

After intrauterine fetal death, women with a scarred uterus are at increased risk of uterine rupture, which should be taken into account when considering options for birth and if induction is carried out [13]. In our study, 3 women presented with complications (2 women presented with hemorrhage and 1 woman presented with hematuria) in the ethacridine lactate group, and two women presented with postpartum hemorrhage secondary to uterine atony with 560 ml and 630 ml of blood loss, respectively. Among 3 women presented with complications, one woman was prior to cesarean section and had induction failure with 630 ml of blood loss. The other two women did not have a history of cesarean section or uterine surgery. The one patient with hematuria had no history of cesarean section or uterine surgery, with normal B-ultrasound scans. The reason remains unclear, and she had a rapid recovery after conservative treatment, including bladder irrigation. In our study, there were four patients who underwent artificial amniotomy (+ oxytocin), among whom two suffered from hemorrhage (850 ml and 1500 ml, respectively) due to placental abruption, and one patient suffered from rectal injury due to macrosomia (4015g) and underwent rectal repair with the help of the surgeon.

There were some strengths in our study. The report of ethacridine lactate used in stillbirth induction in the third trimester has enriched the evidence of stillbirth induction, and we found that < 75mg ethacridine lactate had a similar effect as ≥ 75mg ethacridine lactate in the induction of third-trimester stillbirth. However, several limitations should also be noted. First, this study was a hospital-based retrospective study, and incomplete data for variables might result in statistical bias. Second, the number of patients included in this study was limited, which resulted in difficulties for further analyses. Third, the retrospective design of the study and the lack of uniformity in both the method chosen for induction as well as variations within the method chosen made the comparison of various methods of induction difficult in this study, especially the comparison between ethacridine lactate and misoprostol.

In conclusion, our study confirms that mifepristone combined with ethacridine lactate is a safe and low-risk induction method for patients with third-trimester stillbirths. Prospective or randomized studies should be performed to compare mifepristone combined with ethacridine lactate with misoprostol in third-trimester stillbirth induction and to further examine these conclusions from a clinical perspective.

## Conclusion

Mifepristone combined with ethacridine lactate is a safe and low-risk induction method for patients with stillbirth in the third trimester.

## Data Availability

The data can be available from the corresponding author on the resealable request.

## Declarations

### Author details

^1^International Peace Maternity and Child Health Hospital, School of Medicine, Shanghai Ciao Tong University, Shanghai, China.

^2^Shanghai Key labouratory of Embryo Original Diseases, Shanghai 200030, China;

^3^ Institute of Birth Defects and Rare Diseases, School of Medicine, Shanghai Ciao Tong University, Shanghai 200030, China.
